# Further to the Left: Stress-Induced Increase of Spatial Pseudoneglect During the COVID-19 Lockdown

**DOI:** 10.3389/fpsyg.2021.573846

**Published:** 2021-02-24

**Authors:** Federica Somma, Paolo Bartolomeo, Federica Vallone, Antonietta Argiuolo, Antonio Cerrato, Orazio Miglino, Laura Mandolesi, Maria Clelia Zurlo, Onofrio Gigliotta

**Affiliations:** ^1^Natural and Artificial Cognition Laboratory, Department of Humanities, University of Naples Federico II, Naples, Italy; ^2^Sorbonne Université, Inserm U 1127, CNRS UMR 7225, Paris Brain Institute, ICM, Hôpital de la Pitié-Salpêtrière, Paris, France; ^3^Dynamic Psychology Laboratory, Department of Political Sciences, University of Naples Federico II, Naples, Italy; ^4^Department of Humanities, University of Naples Federico II, Naples, Italy; ^5^Institute of Cognitive Sciences and Technologies, National Research Council, Rome, Italy

**Keywords:** cognition, psychology, stress, pseudoneglect, coronavirus, quarantine, pandemic

## Abstract

**Background:**

The measures taken to contain the coronavirus disease 2019 (COVID-19) pandemic, such as the lockdown in Italy, do impact psychological health; yet, less is known about their effect on cognitive functioning. The transactional theory of stress predicts reciprocal influences between perceived stress and cognitive performance. However, the effects of a period of stress due to social isolation on spatial cognition and exploration have been little examined. The aim of the present study was to investigate the possible effects and impact of the COVID-19 pandemic on spatial cognition tasks, particularly those concerning spatial exploration, and the physiological leftward bias known as pseudoneglect. A right-hemisphere asymmetry for spatial attention processes crucially contributes to pseudoneglect. Other evidence indicates a predominantly right-hemisphere activity in stressful situations. We also analyzed the effects of lockdown on coping strategies, which typically show an opposite pattern of hemispheric asymmetry, favoring the left hemisphere. If so, then pseudoneglect should increase during the lockdown and be negatively correlated with the efficacy of coping strategies.

**Methods:**

One week before the start of the lockdown due to COVID-19 in Italy (T1), we had collected data from a battery of behavioral tests including tasks of peri-personal spatial cognition. During the quarantine period, from late April to early May 2020 (T2), we repeated the testing sessions with a subgroup of the same participants (47 right-handed students, mean age = 20, SD = 1.33). At both testing sessions, participants performed digitized neuropsychological tests, including a Cancellation task, Radial Arm Maze task, and Raven’s Advanced Progressive Matrices. Participants also completed a newly developed COVID-19 Student Stress Scale, based on transactional models of stress, and the Coping Orientation to Problems Experienced—New Italian Version (COPE-NIV) to assess coping orientation.

**Results:**

The tendency to start cancelation from a left-sided item, to explore first a left-sided arm of the maze, and to choose erroneous response items on the left side of the page on Raven’s matrices increased from T1 to T2. The degree of pseudoneglect increment positively correlated with perceived stress and negatively correlated with Positive Attitude and Problem-Solving COPE-NIV subscales.

**Conclusion:**

Lockdown-related stress may have contributed to increase leftward bias during quarantine through a greater activation of the right hemisphere. On the other hand, pseudoneglect was decreased for better coping participants, perhaps as a consequence of a more balanced hemispheric activity in these individuals.

## Introduction

Coronavirus disease 2019 (COVID-19) broke into a worldwide pandemic (World Health Organization, 2020) at the beginning of 2020. At the time of writing, there are more than 7.5 million confirmed cases throughout 215 countries, with more than 400,000 deaths. Italy was the first European Union (EU) country to be hit by a dramatic COVID-19 outbreak, with a quick and heavy impact on public physical and psychological health. Millions of people have experienced an abrupt change in their lives, due not only to the spreading of the illness but also to the measures put in place to prevent the contagion and limit the outbreak. On March 9, 2020, the Italian government imposed a national quarantine and several consequent lockdown restrictions, which ended on May 4, 2020, for some activities and on May 18, 2020, for others. Thus, the Italian quarantine lasted 70 days.

Brooks et al. (2020) examined previous research on the psychological impact of quarantine and reported a high prevalence of symptoms of psychological distress and disorder: especially low mood and irritability, but also emotional disturbance, depression, stress, insomnia, post-traumatic stress symptoms, etc. Moreover, they found that the effects of security measures due to a pandemic can affect not only short-term but also long-term psychological state (Brooks et al., 2020). Poor sleep quality, for example, can affect mental health (Gehrman et al., 2013; Franceschini et al., 2020) and alter emotional and cognitive functioning (Altena et al., 2020). As emphasized by the American Psychological Association (Novotney, 2020), social isolation can trigger several health risks. Feeling isolated can lead to poor sleep, poor cardiovascular health, depressive symptoms, and impaired executive function. These effects tend to impair the ability to stay focused, emotional control, retrieval of information, and the capacity to follow directions. Even brief periods of loneliness and isolation can have negative consequences on both physical and mental well-being (Cacioppo and Patrick, 2008).

Restrictions could affect different life domains among students, inducing specific perceived stressors related to academic studying, relationships with university colleagues, relationships with professors, social isolation, risk of contagion, relationships with relatives, and sexual life (Zurlo et al., 2020). Moreover, restrictions could also affect physical activity, socializing (except virtual social media), proper nutrition, and good quality of sleep. These restrictions were likely to result in increased stress and psychological disease (Mandolesi et al., 2018; Brooks et al., 2020; Zurlo et al., 2020) as well as in recourse to coping strategies to deal with it. However, less is known about the potential impact of quarantine on spatial cognition, a heterogenous set of processes incorporating spatial memory (Mandolesi et al., 2009b; Sorrentino et al., 2019), egocentric and allocentric representation and mapping abilities (Klatzky, 1998; Foti et al., 2020), and visuo-perceptive abilities, including spatial attention (Newcombe and Huttenlocher, 2007; Bartolomeo et al., 2012; Newcombe, 2018; Bartolomeo and Malkinson, 2019; Bartolomeo, 2020).

The transactional theory of stress predicts a reciprocal influence between perceived stress and cognitive performance and underlines the key role played by individual differences, such as coping strategies, in influencing this relationship (Lazarus and Folkman, 1984; Matthews et al., 2000). The individual adaptation process to a significant source of stress (such as the current COVID-19 pandemic lockdown) consists of appraisals of primary control (i.e., perceived possibilities to modify the situation to reduce its negative impact) and secondary control (i.e., perceived possibilities to modify the appraisal of circumstances to achieve a positive adjustment).

Thus, similar sources of stress may have a different subjective impact. Stress is a dynamic concept, depending on the constant interplay between individual and situational factors that reciprocally influence each other, and the potential efficacy of the different coping strategies adopted to deal with perceived stress and to enhance adjustment is strongly situation-specific and related to the interaction between the individuals and the situations (Zurlo et al., 2013, 2019). From this perspective, in particular, a perceived lack of controllability can lead to lower levels of performance (Matthews and Campbell, 2009).

Little is known about the effects of a period of stress (such as quarantine and social isolation) on spatial cognition. Animals being exposed to chronic stress show impaired exploratory behavior (Brydges et al., 2012; ter Horst et al., 2012; van der Kooij et al., 2018). Rats exposed to chronic stress in their early life show atypical leftward asymmetry in turning behavior (Mundorf et al., 2020). In particular, stress might play different roles at different stages of development: early exposition might lead to structural brain changes, whereas later exposition might modulate functional aspects (Berretz et al., 2020).

Gruzelier and Phelan (1991) found that stress was able to shift the hemispheric balance in a divided visual field lexical task toward the left visual field in a sample of medical students. Richardson and VanderKaay Tomasulo (2011) induced stress in human participants by using the frustrating Star Mirror Tracing Task and found slower spatial responses in a navigation task and a perspective taking task, as compared with non-stressed control participants. However, Schwabe et al. (2007) found no evidence of an effect of stress on spatial learning, and Duncko et al. (2007) found improved performance on a virtual navigation task after hand immersion in ice water (cold pressor stress).

Directional spatial effects offer a possibility to quantify the effects of stress on spatial cognition. A basic, evolutionarily conserved pattern of asymmetry sees the right hemisphere taking control of responses to novel, unpredicted and potentially dangerous changes in the environment (Compton et al., 2000; Vallortigara and Versace, 2017; Bartolomeo and Malkinson, 2019). Another, well-known pattern of asymmetry favoring the human right hemisphere concerns the fronto-parietal brain networks important for orienting and control of spatial attention (Corbetta et al., 2008; Bartolomeo and Malkinson, 2019). A relative hyperactivity of right-hemisphere attention networks might push spatial attention leftward. This directional attention bias contributes to a small, physiological leftward bias in spatial processing (Toba et al., 2011), labeled pseudoneglect (Bowers and Heilman, 1980). Pseudoneglect can manifest itself during the bisection of horizontal lines, as a small leftward deviation of the subjective midpoint (Jewell and McCourt, 2000) or as a bias to start visual search from a left-sided item (Gigliotta et al., 2017).

Evidence on structural and functional brain asymmetries regarding attention networks and stress response (both involving the right hemisphere, see Ocklenburg et al., 2016; Zach et al., 2016; Gigliotta et al., 2017) led us to hypothesize a relation between stress and pseudoneglect. Specifically, two predictions were made: (1) higher level of stress should increase the magnitude of pseudoneglect and (2) effective coping strategies that may preferentially reduce right-hemisphere activation (Lindauer et al., 2008) should reflect in a lower magnitude of pseudoneglect. Just before the beginning of the COVID-19 lockdown, we had assessed visuospatial performances in a group of Italian university students. Thus, we had the unique opportunity to test our predictions by comparing students’ performances before and during the lockdown.

## Materials and Methods

### Participants

Throughout the month of February 2020, before the Italian lockdown, we conducted data collection sessions on peri-personal spatial cognition tasks. The data collection ended on March 2, exactly 1 week before the start of the lockdown due to COVID-19 in Italy. We therefore decided to perform a second data collection with the same participants during the quarantine period. Specifically, the session lasted 2 weeks from late April to early May.

Before the beginning of the lockdown, 102 Psychology and Philosophy students (81 females) of the University of Naples Federico II aged between 18 and 26 years (mean = 19.5, SD = 1.5) voluntarily enrolled in the first experimental session. Selection criteria for participants’ recruitment included normal or corrected-to-normal vision. Students were contacted later on during quarantine, and 55 out of 102 students agreed to participate in the second session. Seven out of 55 participants were excluded because they reported left hand preference. Left-handers were excluded because of evidence of higher performance variability on visuospatial tasks (see, for example, Sampaio and Chokron, 1992) and of decreased pseudoneglect effects (Jewell and McCourt, 2000). One additional participant did not conclude the session because of technical problems. The final sample consisted of 47 right-handed students, aged between 18 and 24 years (mean = 20, SD = 1.33), 41 females and 6 males.

Written informed consent was obtained from all participants. The study was approved by the Local Ethics Committee of the University of Naples Federico II (protocol number: 12/2020) and was carried out in accordance with the Declaration of Helsinki.

### Measures

#### Neuropsychological Tests

##### Cancellation Task

In the present study, we administered a digitized Cancellation task developed by Gigliotta et al. (2017). Each trial starts with participants touching (or clicking on) a green button located at the center of the screen. Participants are then presented with five round red stimuli randomly arranged on an electronic screen. They have to cancel all the stimuli as fast as possible with a stylus pen touch or a mouse click (depending on the user interface). The canceled item changes in color to a brighter nuance of red. Thirty trials were administered, with randomly different spatial disposition of targets.

##### Radial Arm Maze Task

The Radial Arm Maze (RAM; Olton and Samuelson, 1976) consists of a central area with identical radiating arms. It is extensively used to assess the spatial abilities of laboratory rodents and human participants (Overman et al., 1996; Mandolesi et al., 2009a,b; Foti et al., 2011, 2020). The aim is to recover rewards hidden at the end of each arm. Different strategies can be implemented (for example, visit a specific sequence of arms, adjacent, opposite, or alternating, etc.). We used a digitized version (Mandolesi and Gigliotta, submitted) whereby participants control a ladybug, positioned in the center of the labyrinth, along the arms to retrieve hidden ladybugs placed at the end of each arm. There were six trials, with a time limit of 60 s per trial. The number of arms gradually increased over trials from 3 to 8 arms. In the present work, we analyzed results from the 8-arm maze, the condition with the highest spatial resolution.

##### Raven’s Advanced Progressive Matrices

The Raven’s Advanced Progressive Matrices (APM; Raven et al., 1962) are used to assess non-verbal and “fluid” intelligence and require the direct analysis, construction, and integration of a series of visual items. Raven’s matrices questions consist of visual geometric designs with a missing piece. Participants are asked to choose the missing piece between eight alternatives, arranged along four vertical columns disposed from the left to the right of the page below the test image. A digital version of the Raven’s APM was administered in the present study, for which the matrices were transposed on Google Modules. Only set I (12 items with 8 possible responses) was administered.

Costa et al. (1969) administered the Raven’s APM to patients suffering from left neglect after right-hemisphere damage and assessed the spatial side (left or right) of the error responses. The results showed that patients tended to erroneously choose right-sided items. Colombo et al. (1976) administered Raven’s APM to patients with left and right brain injuries and found that patients tended to prefer ipsilesional candidate items. This position preference was especially evident in patients with right-hemisphere damage. This evidence, suggesting that spatial biases can influence performance on the Raven’s APM, incited us to employ such a space-based assessment in the present setting.

### Questionnaires

#### The COVID-19 Student Stress Questionnaire

The COVID-19 Student Stress Questionnaire (CSSQ; Zurlo et al., 2020) was specifically developed to assess university students’ perceived stress during the COVID-19 pandemic lockdown. It consists of 7 items on a 5-point Likert scale ranging from 0 (“not at all stressful”) to 4 (“extremely stressful”). For the purpose of instrument design, perceived stress was operationalized based on transactional models of stress (Lazarus and Folkman, 1984). Each item was developed to cover different domains that could have been subject to variations due to the COVID-19 pandemic lockdown and, therefore, that may be potentially perceived as sources of stress (i.e., risk of contagion; social isolation; relationship with relatives; relationship with colleagues; relationship with professors; academic studying; couple’s relationship, intimacy, and sexual life). The scale provides a Global Stress score ranging from 0 to 28. The CSSQ was developed and tested in a sample of 514 Italian university students, and it was confirmed to be a valid and reliable measure. The Global Stress score revealed significant correlations, in the expected directions, with measures of Anxiety (*r* = 0.55, *p* < 0.01), Depression (*r* = 0.56, *p* < 0.01), and Somatization (*r* = 0.39, *p* < 0.01), as assessed by means of the Symptom Checklist-90—Revised (SCL-90-R; Prunas et al., 2010). The questionnaire revealed a satisfactory internal consistency (Cronbach’s alpha = 0.71).

The results of the CSSQ scale validation study highlighted the presence of three significant factors, which the authors labeled as: 1) “Relationships and Academic Life,” which comprised the four items covering perceived stress related to relationships with relatives, relationships with colleagues, relationships with professors, and academic studying; 2) “Isolation,” which comprised the two items exploring perceived stress related to social isolation and changes in sexual life due to the containment measures; and 3) “Fear of Contagion,” which comprised the item assessing perceived stress related to the risk of infection. Therefore, we decided to analyze any relationships between the increase in left bias and the stress measured through the CSSQ scale.

#### Coping Orientation to Problems Experienced—New Italian Version (COPE-NIV; Sica et al., 2008)

The questionnaire consists of 60 items on a 5-point Likert scale ranging from 1 (“I usually don’t do this at all”) to 4 (“I usually do this a lot”) divided into five subscales: Seeking Social Support (12 items covering strategies centered on seeking support for instrumental or emotional reasons and focusing on and venting of emotions; Cronbach’s α = 0.88), Avoiding (16 items covering strategies centered on detaching, denial, humor, alcohol and drug disengagement, behavioral disengagement, and mental disengagement; Cronbach’s α = 0.70), Positive Attitude (12 items covering strategies centered on positive reinterpretation and restraint coping; Cronbach’s α = 0.76), Problem Solving (12 items covering strategies centered on suppression of competing activities, planning, and active coping; Cronbach’s α = 0.83), and Turning to Religion (8 items covering strategies centered on seeking comfort in religious and spiritual practices; Cronbach’s α = 0.85).

### Procedure

#### Pre-Lockdown Session (T1)

The first experimental session took place in a quiet room of the University of Naples Federico II. In the room, there was a large table with chairs around it; on the table, there were an 8-inch tablet to be used by participants and a computer in front of the experimenter. Participants sat in front of the experimenter. The total time to complete all tests was around 20 min.

The first session test battery we administered included, among other tests, the Cancellation task, the RAM task, and the Raven’s APM task. The Cancellation task and the RAM task were administered by means of specific software running on an 8-inch tablet and performed using a stylus pen to interact with the screen. Participants were comfortably seated with a viewing distance of ∼40 cm, and the tablet was placed on the table in front of them in a vertical position.

A digital version of the Raven’s APM was first administered, for which the matrices were transposed on Google Modules. Participants used a 14-inch PC and a mouse to perform the APM. Then, the Cancellation task was administered on the tablet. The instructions were as follows: “As soon as you select the green button with the pen you will see little circles, which you will have to select all in the shortest possible time. If you happen to touch the white screen it will turn black for a moment, then you go on.” Finally, the digitized RAM task was performed by participants. The instructions were as follows: “The objective of this task is to explore all the arms of the mazes, dragging the ladybug there, and find the ladybugs hidden under the jars within 60 s. Remember that you must always go to the center before moving from one arm to another. The first item is for practice.”

#### Lockdown Session (T2)

After about 2 months from the start of the lockdown, we contacted the participants of the first experimental session. A subgroup of 47 students agreed to participate in the second experimental session. The procedure consisted in the administration of the questionnaires, then in the online administration of the Cancellation task, and the digitized RAM task to the participants, through Microsoft^®^ Teams, a unified communication and collaboration platform that combines chat, teleconferencing, content sharing, and application integration.

Participants were asked to fill out the questionnaires on Microsoft^®^ Forms, an online survey maker software. A few days after completing the questionnaires, one experimenter started the cognitive test sessions and carried out the online meetings on Microsoft^®^ Teams platform, with each student separately. The experimenter first explained the test procedure methods, ensuring that both network connections were working properly. The software implementing the spatial tasks ran on the experimenter’s computer. After giving the same task instructions as in the pre-lockdown session, the experimenter activated the Microsoft^®^ Teams platform’s screen sharing mode, so that the participants had the control of the experimenter’s screen and were able to carry out the tasks. Thus, there were minor differences in user interface between T1 (touch stylus used for the Cancellation task and the RAM task) and T2, when the mouse was instead used for all tests.

### Parameters

In both the experimental sessions, the following parameters were analyzed.

For the Cancellation task, we first defined the center of the display as 0, so that the values of the X pixel coordinates assumed a negative sign for the left side of the screen and a positive sign for the right side. Then, we calculated the average position on the x-axis of the first canceled stimulus for each participant (see Gigliotta et al., 2017, for a detailed description of the procedure). In order to assess potential differences in spatial bias before (T1) and during (T2) the lockdown, we calculated the increment of leftward preference, in canceling the first stimuli, from T1 to T2.

For the RAM task, we defined as 0 the center of the display. The values of the X pixel coordinates were negative for the left side of the screen and positive for the right side. We focused on the performance on the 8-arm maze, which offered participants the largest number of potential exploration strategies. We assessed the coordinates of the first explored arm for the 8-arm maze, as well as the spatial sequence of the visited arms.

For Raven’s APM, in addition to the test scores, we obtained a measure of position preference (see Costa et al., 1969), by assessing the location in space (left or right) of the error responses chosen below the target figure of each matrix. Therefore, we calculated the average number of items erroneously chosen for each side of space, among the four left-sided and the four right-sided alternatives.

## Results

Data analysis was run on JASP (https://jasp-stats.org/), version 0.12.2.

### Cancellation Task

First, we investigated the presence of a lateralization of the first canceled stimulus in the Cancellation task: results showed a left-biased distribution of the first canceled stimulus both for T1 (Wilcoxon–Mann–Whitney two-tailed test, *Z* = −103, *p* < 0.001; mean = −60.1, SD = 62.33) and for T2 (Wilcoxon–Mann–Whitney two-tailed test, *Z* = −18, *p* < 0.001; mean = −83.46, SD = 49.97), thus confirming the previously reported tendency to start the visual search from a left-sided target (pseudoneglect) on this task (Gigliotta et al., 2017).

**FIGURE 1:**
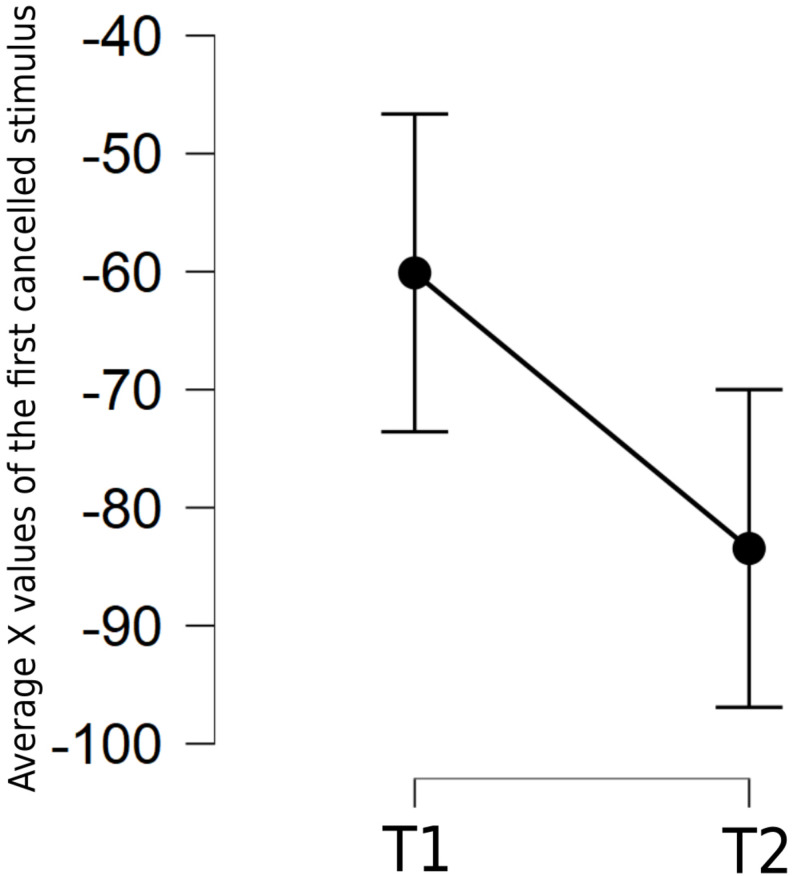
Mean of X values of the first canceled stimulus in the Cancellation task at T1 and T2. Error bars indicate 95% confidence intervals.

Then, a repeated measure ANOVA was conducted on the spatial X coordinates of the first canceled stimulus for each trial in T1 and T2, to evaluate potential lockdown-induced changes in patterns of spatial exploration. The independent variable was the time of testing (T1, T2); the dependent variable was the coordinate (in pixels) of the first canceled stimulus. Figure 1 shows that, on average, the first canceled stimulus at T2 was 23 pixels further to the left (mean = −83.46 pixels, SD = 49.97) as compared with its position at T1 [mean = −60.10, SD = 62.33, *F*(1,46) = 6.10, *p* = 0.017, with a moderate sample size effect, η^2^ = 0.117].

**FIGURE 2:**
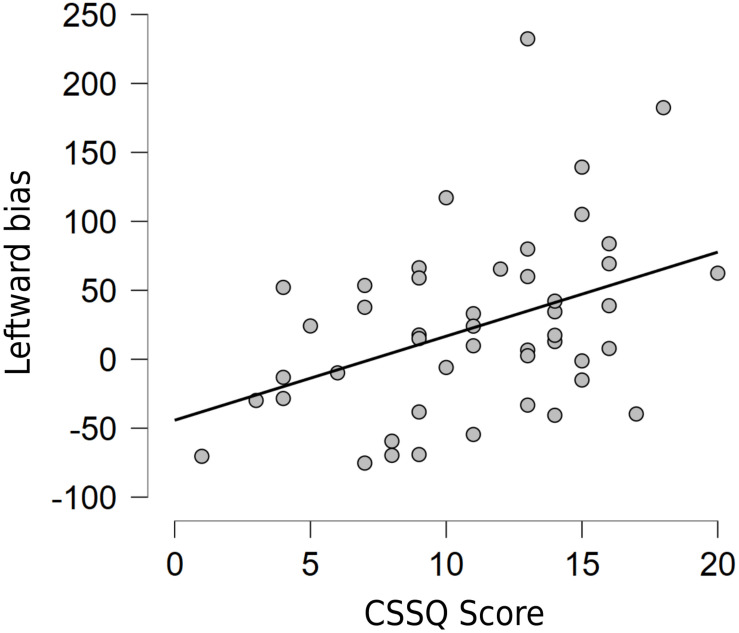
Relationship between the CSSQ scale global score and leftward bias variation in the Cancellation task from T1 to T2.

To further assess the potential relationship between stress and magnitude of pseudoneglect, we conducted a two-tailed Pearson’s correlation analysis between the results of the CSSQ and the pseudoneglect increment at T2 from T1. The null hypothesis was that the two variables are not related in our sample; conversely, the alternative hypothesis was that the stress and the magnitude of pseudoneglect are related. The results showed a significant correlation between the CSSQ scale and the leftward biased exploration of the space (*r* = 0.407, *p* = 0.004), so we could accept the alternative hypothesis and reject the null one: particularly, as stress levels increase, the exploration bias to the left seems to be also accentuated (Figure 2).

**FIGURE 3:**
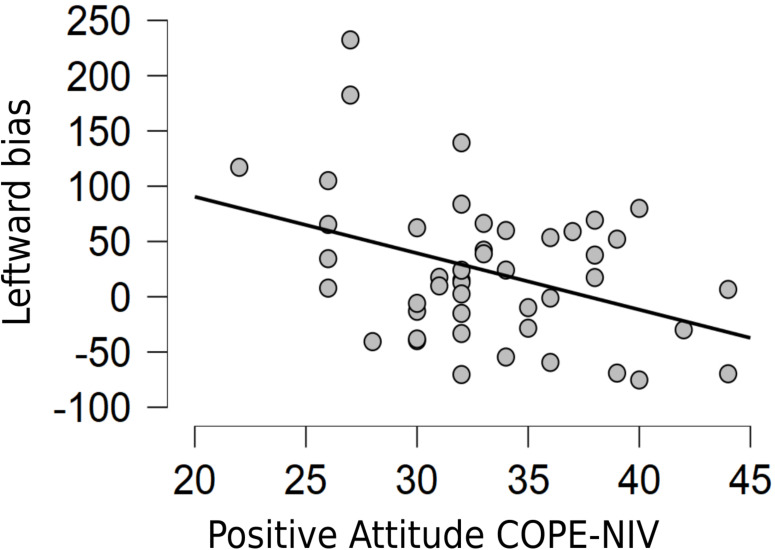
Relationship between Positive Attitude COPE-NIV subscale and leftward bias variation in the Cancellation task from T1 to T2.

**FIGURE 4:**
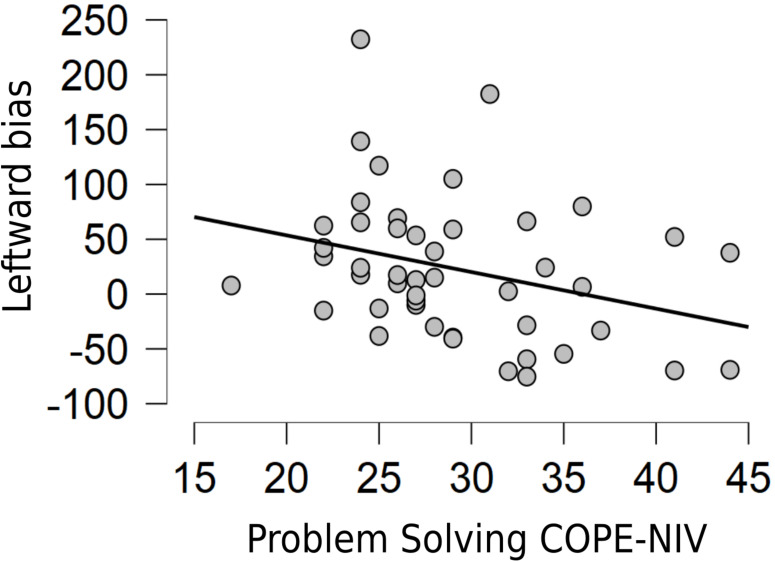
Relationship between Problem Solving COPE-NIV subscale and leftward bias variation in the Cancellation task from T1 to T2.

A second two-tailed Pearson’s correlation analysis investigated the potential relationship between coping (assessed through the five subscales of the COPE-NIV) and pseudoneglect increment at T2. The results showed a significant correlation between 2 out of 5 COPE-NIV subscales (Positive Attitude and Problem Solving) and lower leftward biased exploration of the space (*r* = −0.385, *p* = 0.008 and *r* = −0.308, *p* = 0.037, respectively; see Figures 3, 4). This correlation indicates that pseudoneglect decreases with increasing active coping strategies. Instead, no significant correlation resulted between the other COPE-NIV subscales, Seeking Social Support (*r* = 0.100, *p* = 0.507), Avoiding (*r* = −0.072, *p* = 0.633), and Turning to Religion (*r* = −0.116, *p* = 0.445).

### Digitized RAM Task

**FIGURE 5:**
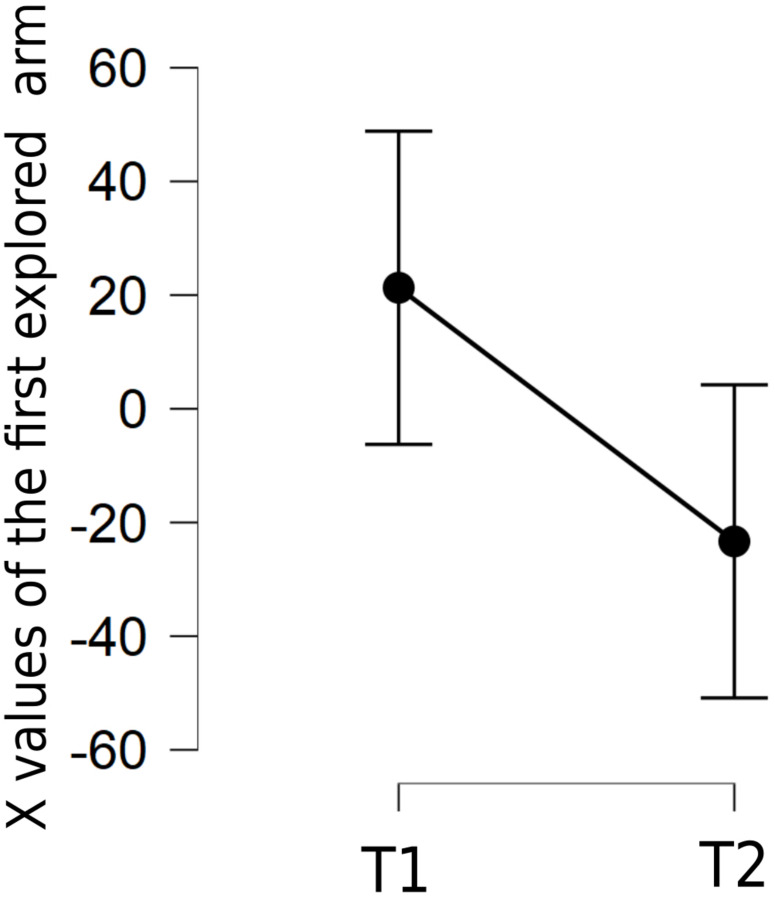
Mean of X values of the first explored arm on the digitized RAM task at T1 and T2. Error bars indicate 95% confidence intervals.

We investigated the lateralization of the first explored arm of the RAM and verified if there was a significant variability of the lateralization between T1 and T2. A repeated measures ANOVA on the x coordinates of the first arm (of the 8-arm maze) chosen indicated that participants tended to start exploration from a left-sided arm at T2 (mean = −23.04, SD = 112.74), whereas they preferred to start from a right-sided arm at T1 [mean = 21.62, SD = 110.47, F(1,46) = 5.31, *p* = 0.03, η^2^ = 0.103] (Figure 5).

Then, to assess a potential relationship between stress/coping and the deviation of the maze exploration to the left side at T2, a two-tailed Pearson’s correlation analysis between the results of the CSSQ/COPE-NIV and the laterality variation at T2 from T1 was conducted. The results do not show any significant correlation of the variation in lateralization, neither with the global score of the CSSQ scale (*r* = −0.173, *p* = 0.246) nor with the CSSQ subscale scores, nor with the 5 COPE-NIV subscales, such as Positive Attitude and Problem Solving coping strategies (*r* = −0.092, *p* = 0.545 and *r* = −0.019, *p* = 0.899, respectively).

### Raven’s APM

Participants obtained scores in the normal range both at T1 (mean = 10.085, SD = 1.851) and at T2 (mean = 9.745, SD = 1.750). There was no significant effect of time of testing on accuracy [repeated measures ANOVA on the correct answers, *F*(1,46) = 2.012, *p* = 0.163].

**FIGURE 6:**
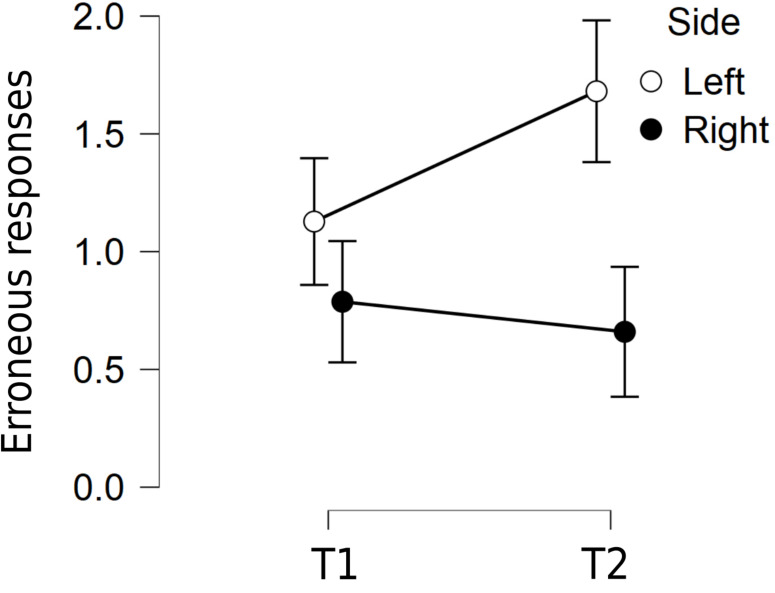
Erroneous responses to the Raven’s Advanced Progressive Matrices at T1 and T2 for the right and left sides. Error bars indicate 95% confidence intervals.

To assess changes in position preference for erroneous responses (Costa et al., 1969), we conducted a 2 (period of testing: T1, T2) × 2 (error side: right, left) repeated measures ANOVA. There was a main effect of error side: *F*(1,46) = 22.41, *p* < 0.001, η^2^ = 0.14, because participants showed a bias to choose a left-sided item (mean = 1.40, SD = 1.30) over a right-sided one (mean = 0.72, SD = 1.01). Time of testing approached significance (*p* = 0.06), because participants tended to make more errors during quarantine (mean = 1.17) than before it (mean = 0.96). Importantly, the two factors interacted *F*(1,46) = 4.91, *p* = 0.032, η^2^ = 0.036, because the leftward bias increased during quarantine (Figure 6).

Finally, to assess a potential relationship between stress/coping and the deviation of Raven’s errors to the left side at T2, a two-tailed Pearson’s correlation analysis between the results of the CSSQ/COPE-NIV and the laterality variation at T2 from T1 was conducted. The results do not show any significant correlation of the variation in lateralization, neither with the global score of the CSSQ scale (*r* = −0.017, *p* = 0.910) nor with the CSSQ subscale scores, nor with the 5 COPE-NIV subscales, such as Positive Attitude and Problem Solving coping strategies (*r* = −0.148, *p* = 0.327 and *r* = −0.079, *p* = 0.604, respectively).

## Discussion and Conclusion

The aim of this research was to assess whether the stressful conditions experienced by students during the harsh quarantine measures taken in Italy (a country strongly hit by COVID-19) had any influence on their spatial cognition abilities. The results indicated several indices of such an influence.

Specifically, we found a significant leftward shift in three tasks tapping on spatial abilities from T1 (pre-lockdown) to T2 (circa after 2 months of harsh quarantine). The tasks were (a) the Cancellation task, (b) the digitized RAM task, and (c) the Raven’s APM task. Importantly, there were no changes in the general accuracy on the Raven’s APM task, a test of general non-verbal intelligence; the only time-related change was an increase of leftward spatial bias in the choice of an (erroneous) response item. Thus, the time-related changes we observed seem to be relatively specific to the spatial domain. A potential confound could be the mode of response used for the Cancellation task. Participants used a touch pen at T1 and a computer mouse at T2. However, a similar increase in leftward bias with time also occurred on the Raven’s matrices, where participants always responded by using the mouse.

Were these changes in spatial bias really related to lockdown-induced stress? Evidence supporting this possibility comes from (1) the positive correlation between stress measured through the CSSQ scale and the increase of pseudoneglect during the lockdown and (2) the negative correlation that we observed between the increment of pseudoneglect and specific active coping strategies, which mirrored the positive correlations between pseudoneglect and time of testing on the Cancellation task (for the other tasks, we should take into account differences on the cognitive functions they rely on and different measures of stress). Individuals who were able to resort to positive attitude and problem-solving coping strategies displayed lesser leftward bias than those who obtained higher scores in perceived stress.

Our findings seem, therefore, in line with research underlining the impact of perceived stress on individuals’ performance (Matthews et al., 2000; Matthews and Campbell, 2009). They also provide new evidence supporting the efficacy of the adoption of strategies centered on activity and positive reappraisal (Santarnecchi et al., 2018; Zurlo et al., 2019).

The neurobiological underpinnings of physiological leftward bias (pseudoneglect) are likely to rely on hemispheric asymmetries of attention networks (Corbetta et al., 2008; Bartolomeo and Malkinson, 2019). Shifts in line bisection strictly depend on activity in these fronto-parietal networks (Thiebaut de Schotten et al., 2005). For example, activity in the right ventral attention network seems to correlate with the effect of line length in pseudoneglect (Benwell et al., 2014). In a simulation study, Gigliotta et al. (2017) demonstrated that different patterns of asymmetries in artificial attention networks can lead to different levels of pseudoneglect in neuroagents (robots provided with a simulated brain) performing a Cancellation task similar to the one used here.

On the other hand, abundant evidence suggests a relation of stress-related mechanisms with the right hemisphere (Compton et al., 2000; Ocklenburg et al., 2016; Bartolomeo and Malkinson, 2019). Moreover, early life exposure to stress has been proposed as a determinant of psychiatric and neurodevelopmental diseases characterized by atypical brain asymmetries (Berretz et al., 2020). Finally, a recent study on turning behavior in rats highlighted a leftward shift in turning preferences in a group of animals exposed to stressful conditions during the early stage of their lives compared with a control group (Mundorf et al., 2020). Acute and chronic stress can thus affect lateralized behavior in humans and animals, as a result of higher right-hemisphere activation (Ocklenburg et al., 2016). In addition, stress in university students was found to increase connectivity in the attention networks, particularly in the right hemisphere (Soares et al., 2013). Over time, this functional modulation might translate into structural plastic changes. For example, Brem et al. (2020) performed white matter tractography on a group of volunteers after 520 days of confinement and found a general reduction in fractional anisotropy in the right temporo-parietal junction. Coping strategies, on the contrary, might be related to greater left hemisphere connectivity. Santarnecchi et al. (2018) found a positive correlation with the connectivity of the left angular gyrus of performance on the problem-solving subscale of the coping scale used in this study, the COPE-NVI.

The specific relation we found between stress and leftward bias is thus likely to depend at least in part on stress-induced increased activity of right-hemisphere attention networks. The right hemisphere may also facilitate stress hormone responses through the hypothalamic–pituitary–adrenal gland axis (Sullivan, 2004), whereas the left hemisphere structures, such as the medial prefrontal cortex, may increase resilience to stress and control its effects on social behavior (Lee et al., 2015).

The present study was conducted on a relatively limited participant sample (*N* = 47). As a consequence of the strict lockdown measures, the test conditions could not be fully controlled at T2. Despite these limitations, our results linking stress and leftward bias were consistent over several tests. More “ecological” tests of spatial cognition, closer to everyday life activities than the tests we employed here (Cerrato et al., 2019, 2020), may be useful to further assess these relationships. Studies in animals, as well as on simulated neurorobots (Broz et al., 2014; Gigliotta et al., 2015a,b, 2017; Pacella et al., 2017), might further illuminate the intimate mechanisms that link stress to spatial attention.

## Data Availability Statement

The raw data supporting the conclusions of this article will be made available by the authors, without undue reservation.

## Ethics Statement

The studies involving human participants were reviewed and approved by the Local Ethics Committee of the University of Naples Federico II (protocol number: 12/2020). The patients/participants provided their written informed consent to participate in this study.

## Author Contributions

OG, LM, MZ, and PB contributed to the conception and design of the study. FS, FV, AA, and AC collected the data and wrote the first draft of the manuscript. OG performed the statistical analysis. All authors contributed to manuscript revision, and read and approved the submitted version.

## Conflict of Interest

The authors declare that the research was conducted in the absence of any commercial or financial relationships that could be construed as a potential conflict of interest.
